# Modeling and Simulation of Offshore Wind Power Platform for 5 MW Baseline NREL Turbine

**DOI:** 10.1155/2015/819384

**Published:** 2015-10-13

**Authors:** Taufik Roni Sahroni

**Affiliations:** Binus Graduate Program, Bina Nusantara University, Kebon Jeruk Raya No. 27, Jakarta 11530, Indonesia

## Abstract

This paper presents the modeling and simulation of offshore wind power platform for oil and gas companies. Wind energy has become the fastest growing renewable energy in the world and major gains in terms of energy generation are achievable when turbines are moved offshore. The objective of this project is to propose new design of an offshore wind power platform. Offshore wind turbine (OWT) is composed of three main structures comprising the rotor/blades, the tower nacelle, and the supporting structure. The modeling analysis was focused on the nacelle and supporting structure. The completed final design was analyzed using finite element modeling tool ANSYS to obtain the structure's response towards loading conditions and to ensure it complies with guidelines laid out by classification authority Det Norske Veritas. As a result, a new model of the offshore wind power platform for 5 MW Baseline NREL turbine was proposed.

## 1. Introduction

Demand of renewable energy plays an increasingly important role as fossil fuels become more and more expensive and harder to justify in expenses. It is expected that wind energy will contribute 1.1 trillion kilowatt-hours of the total 3.3 trillion kilowatt-hours of renewable energy predicted to be supplied by 2030. Furthermore, it is expected that only sun and wind can provide economical alternative energy sources, as other exotic renewable energy sources remain expensive and unproven [1, 2]. It is apparent that wind and sun are going to be the focus of future engineering efforts. Solar energy is expected to become dominant as Arab countries with vast swaths deserts have already started investigating the prospect of generating solar power to continue developing their countries.

For wind energy, the future lies in developing offshore wind turbines that are capable of harnessing the much higher wind speeds available offshore while avoiding the problem of skyline pollution. A more detailed investigation of the current and future wind energy outlook will be presented in the next section. Wind turbines have progressed vastly since the Dutch first used it to grind their mills and have grown in power, from 25 kW to 2500 kW and more. With the growth of wind turbine size, the tools and engineering expertise used to overcome the challenges of harnessing wind power have also grown and expanded, with computer-aided tools becoming more and more prolific and colleges and universities starting to offer courses on wind technology [3]. Modern offshore wind turbines are now required to generate high-quality, network frequency electricity in an independent and automatic manner and do so for 20 years or more continuously with low to no maintenance in some of the harshest environments in our planet. That will be the challenges facing engineers today.

Initially, wind turbines which rotate on the vertical axis were considered as a design as the expected advantages are omnidirectionality and having gears and power generating equipment located at the base of the tower to lower loads. The shape of the turbine is like an onion, also called the “troposkein curve.” The design failed due to the inherent inefficiencies of the rotor and extra weight in construction as well as serious metal-fatigue problems due to the tension-loaded rotors [3]. However, the design is still viable in low-power applications and can be mounted on the rooftops of buildings.

The one bladed design is structurally the most efficient as all the blade area is concentrated on one blade. However, the design needs to spin at a higher speed (relative to multibladed designs) to generate power and this leads to higher blade and tower loads. This high speed often generates a lot of noise which can be undesirable in onshore designs. The use of a counterbalance to give the structure static balance is also inefficient use of weight. Hence, commercially, one bladed design has been mostly eliminated.

According to Gardner et al. in Wind Energy–The Facts [3], wind turbines utilize stall and pitch regulation in order to limit the rotor power when experiencing high operational wind speeds. Stall regulation is where the speed of the rotor is held constant or approximately constant even under increasing wind speeds. This causes the angle of flow over the blade sections to steepen. In effect, the blades become increasingly stalled and thus limit the power to acceptable levels, without requiring any additional active control. Aerodynamicists were initially shocked at the idea of using stall as a form of limiting power because in flight aerodynamics, stall is often fatal and can cause planes and helicopters to crash. However, it proved to be effective to control overspeeding and the solution is unique to the wind energy industry. The history of offshore wind power generation is fairly recent: the world's first offshore wind park was built in 1986 off the coast of Denmark. Sixteen 55 kW units produced electricity for the 4,000 citizens in nearby Ebeltoft. Following the project's success, similar parks were built off Scandinavian coasts through the late 1990s. Danish and German firms are known for their expertise when it comes to offshore wind, and they will likely benefit from the current boom that offshore wind power is experiencing. Companies and governments are planning to build 25 offshore wind parks in five European countries with a total capacity of some 1,100 MW.

There are still some hurdles offshore wind energy has to overcome, mainly the high costs connected to construction, maintenance, and operation. Offshore wind units are constantly exposed to high winds, salt water, and corrosive sea air, which makes them more vulnerable to damage than land units. There are many engineering challenges faced by offshore wind turbine technology. The harsh environment represents some of the more extreme environments on our planet, and with natural phenomenon such as typhoons and rough seas, building a floating structure that requires no maintenance over the course of decades will require great engineering feat [6].

The offshore wind market today is heavily influenced by technologies developed from offshore oil and gas. The foundation design has become the biggest factor that will affect the performance of offshore wind turbine platform. However, some designs from offshore oil and gas are fundamentally incompatible with offshore wind, such as the use of dynamic positioning. This paper will carry out the platform design of offshore wind turbine and propose a new foundation design from the commonly used Pile foundation being used today. The objective of this project is to create the foundation and perform the finite element analysis on the platform design for 5 MW Baseline NREL turbine.

## 2. Literature Review

**Figure 1:**
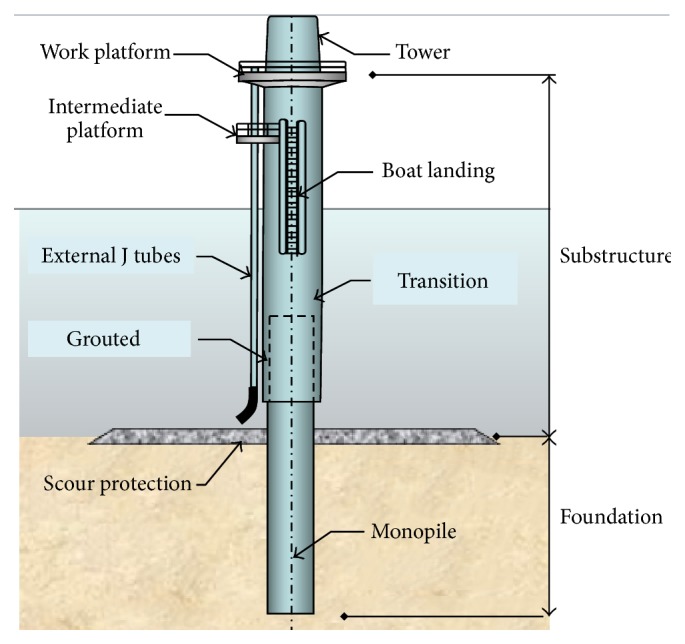
Monopole foundation, consisting of a driven pile [3].

**Figure 2:**
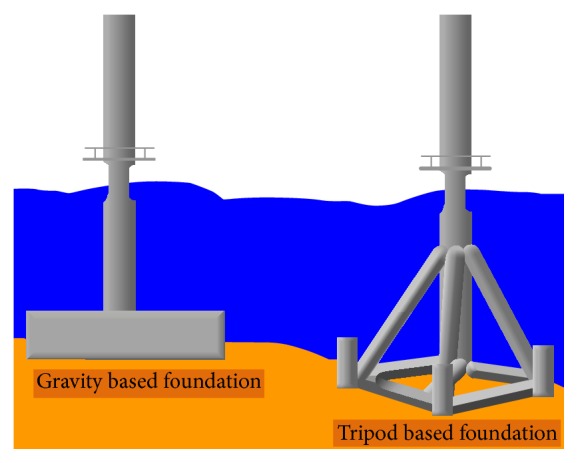
Gravity based and tripod foundation concepts [4].

The foundation or support structure forms the bulk of the OWT installation costs and this is even more so in deep waters. Subroto et al. [11] reported that, for shallow waters somewhere between 20 to 25 meters, monopile and gravity based support structure are dominant and very cost-effective. For transitional waters, the depths are up to 50 meters. Beyond that, the waters are considered deep water for which commercial wind farms have not yet been developed, although prototypes and proof-of-concept designs have been tested out.  Figure 1 shows an overview of the different types of support structure proceeding from shallow to deep waters. Gravity based design is also commonly used consisting of a heavy base connected to the support structure and turbine as shown in Figure 2. However, it is expensive and used only in very shallow waters. The tripod is another preferred design, and many different configurations have been tested with varying geometries and also with and without driven piles for each leg.

**Figure 3:**
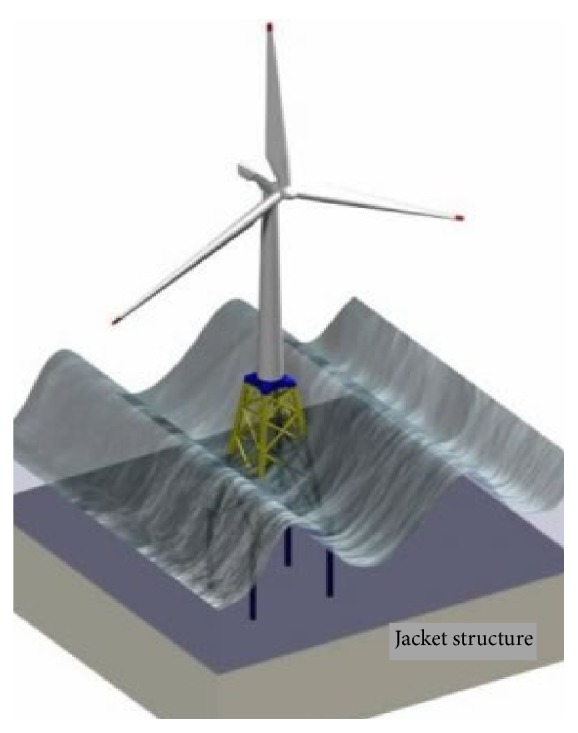
Jacket structure [5].

Figure 3 shows another design which is jacket based. It utilizes a series of tubular joints to form a square tapered structure. This structure is used widely in the offshore oil and gas production. For adoption for OWT use, the jacket design has been studied due to very favorable, as the additional wind loading does not significantly detract from the jacket's torsion strength.

**Figure 4:**
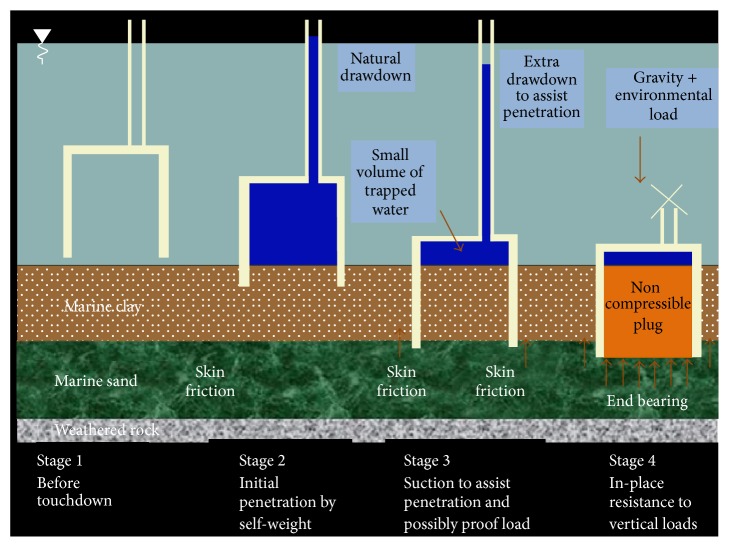
Suction caisson foundation installation stages [6].

It has been demonstrated in China [5] and Germany [12], where the environmental loads are quite extreme with ice and earthquake in the case of China and very deep waters of 50 m and large 5 MW turbines in the case of Germany. The suction caisson foundation is the leading candidate for Hong Kong's own offshore wind turbine farm [6] as it is environmentally friendly and installation does not disturb the surrounding marine life as shown in Figure 4. It is simply lowered into the site together with a powerful pump. Once settled, the pump will extract water, creating a pressure difference which forces the caisson into the seabed. Once completed, the caisson forms a powerful seal at the seabed due to the pressure difference, and the pump is undocked.

**Figure 5:**
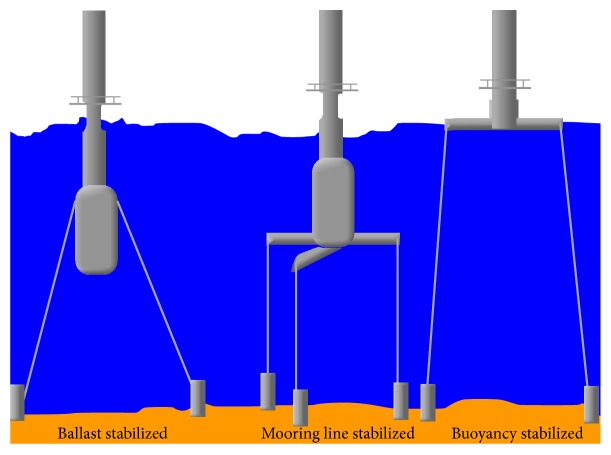
Several structures proposed for transition water depths [4].

Caissons can be combined with guy wires and monopole into a guyed pile caisson [13] design although it has not been explored yet for use with OWT.  Figure 5 shows the three structures shown above are three main concepts to provide stability in transitional water depths. They are shown stabilized using catenary or tension lines and permit some amount of movement; thus, these structures are also known as* compliant* towers. However, not all of the concepts require stabilizing lines.

Butterfield [14] elaborates on the three concepts. The first is ballast stabilized, where large tanks called ballast are hung below a turbine to provide a righting moment with high inertial resistance to pitch and roll. Mooring lines help stabilize the structure. The second is also known as a Tension Leg Platform, where tension in the mooring lines helps provide the righting moment. Finally, the buoyancy stabilized concept achieves stability through use of distributed buoyancy; this principle is demonstrated in barges. Again as mentioned earlier, the use of tension lines for stability is optional and depends on the size of the structure and environmental loads. All commercial concepts being explored for stability are hybrids of these three main designs, exploiting the advantages of all three methods to gain static stability.

**Figure 6:**
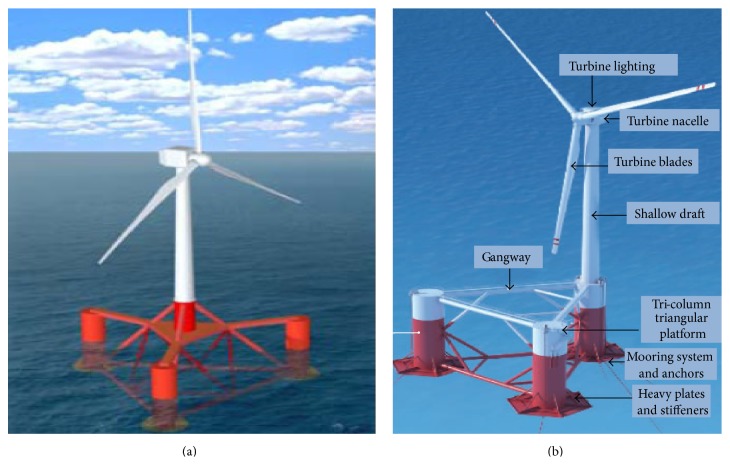
(a) Dutch Tri-Floater [7] and (b) WindFloat semisubmersible concept [8].

Figure 6(a) shows an example: Dutch Tri-Floater has distributed buoyancy tanks attached to the central tower through truss arms. This achieves stability primarily through weighted water plane area (buoyancy) but weight of the steel tanks and truss structure will also provide significant mass to resist overturning moments (ballast). The catenary moorings provide some additional resistance to overturning, mainly due to the mass of the lengthy chain that extends out to a conservative suction pile mooring (mooring line stabilized). As another example, as shown in Figure 6(b), conceived by Marine Innovation & Technology and owned by Principle Power, the WindFloat is a semisubmersible, three-column structure, with a turbine tower, truss, and “water entrapment heave plates” at each column's base, designed to reduce pitch and yaw and make the entire structure more compact. It aims to support deployment of large capacity wind turbines (3.6 MW to 10 MW) in deep water (50 meters or greater).  Table 1 shows the comparison of common foundations used for offshore wind power platform [9].

## 3. Methodology

During the first phase of the project, the main focus is on understanding the field of offshore wind turbine and to gather technical information regarding common installations. The state of computer-aided engineering use in this sector is also gathered. Three key “modules” have been identified in the design and analysis of offshore wind turbines and they are the rotor and blades, the nacelle, and finally the supporting structure. Certification is also important as it represents an industry standard and the design should strive to meet the requirements set by certification authorities. As mentioned earlier, the primary document for reference to this will be DNV's standard. In considering the design, the blade and rotor are a tough subject due to the inherent modeling difficulties encountered even by commercial turbine manufacturers. As for the nacelle, the design will consider the equipment installed, mechanical arrangement, and accessibility. A strategy for reliability and maintainability is also developed.

Finally, the support structure design will follow where necessary the specifications lay out by NREL's 5 MW model offshore turbine. Phase two was focused on development/design of these three components and integration into a CAD model, followed by computer-aided engineering (CAE) analysis on the model loaded by wave to see its response. Postprocessing was used to analyze the data and to visualize the results. The next step is to input the load cases following certification authorities' standard. Then, the solver is run to get the solutions for the problem posed. This is then fed through a postprocessor for a visual representation of results.

The NREL offshore 5 MW baseline wind turbine has been used to establish the reference specifications for a number of research projects supported by the U.S. Department of Energy's Wind Energy Technologies Program. In addition, the integrated European Union UpWind research program and the International Energy Agency Wind Annex XXIII Offshore Code Comparison Collaborative have adopted the NREL offshore 5 MW baseline wind turbine as their reference model. The model has been, and will continue to be, used as a reference by research teams throughout the world to standardize baseline offshore wind turbine specifications and to quantify the benefits of advanced land- and sea-based wind energy technologies [15].

The 5 MW rating is large by today's standards but is assumed to be the minimum rating necessary to make a floating wind turbine system economical because of the large proportion of the costs in the support platform. The wind turbine design is typical of utility-scale, land- and sea-based, and multimegawatt turbines. Based upon many theoretical studies, the gross properties of the theoretical turbine were established as in Table 2 [10].

**Figure 7:**
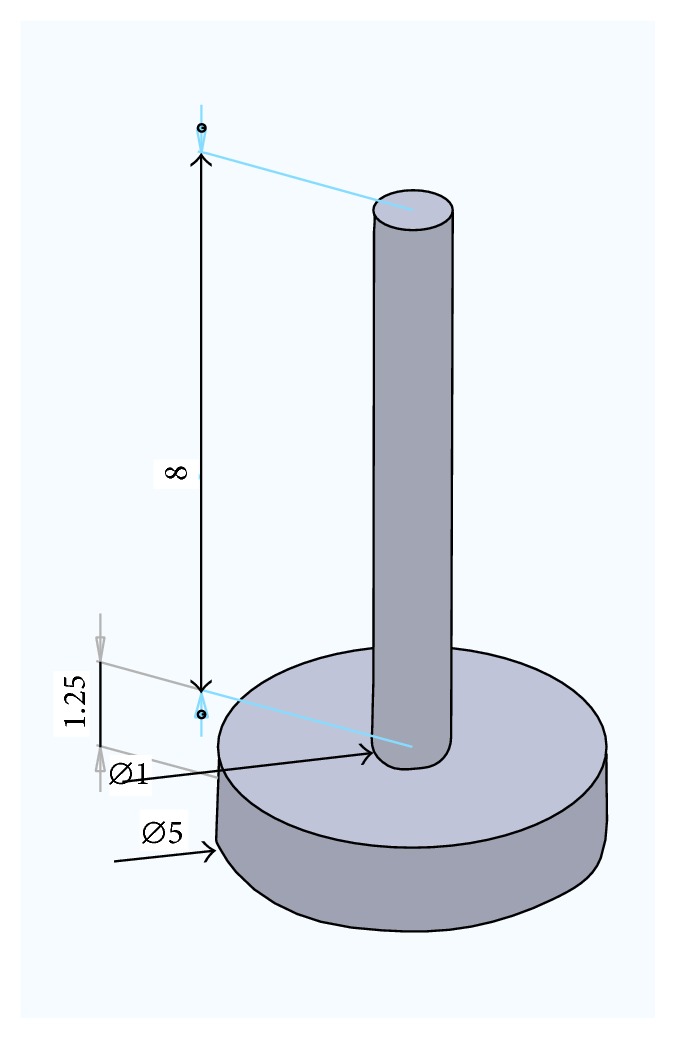
Dimensions for the suction caisson (meters).

Furthermore, the design of the supporting structure must be located in deeper waters where monopole designs are unfeasible.  Figure 7 shows the dimension for the suctions caisson. The detail dimension for the top suctions caisson is 8 m  ×  ⌀1 m and for the bottom suctions caisson is 1 m  ×  ⌀5 m.

The detail design will be guided by reference to OS-J101 standard. First, the material has been identified as special due to the significance of the component in terms of failure consequence and the fact that application of stress condition may increase the probability of brittle fracture. This means the material ultimately selected must come with a Test Certificate EN10204 3.2 with a Category I inspection category for weld inspections. According to Section  10 A101 [10], the requirements for foundation design are applicable only to pile, gravity-type, and stability of sea bottom foundations. Other types not specifically covered will be specially considered. Nonetheless, the specifications will be followed where possible.

For an effective stress stability test, analysis should be carried out on strength parameters of the soil based on laboratory shear strength analysis with pore conditions included. However, this was unable to be performed in order to validate the specific soil characteristics of a site. Hence, values are taken from literature. In this project, the corrosion protection was not included in the simulation. Generally, installing cathodic and coating are the recommended protection method for the outer skin of the structure while enclosed spaces can use biocides. A corrosion allowance is also usually set and depends on the chloride content of the site seawater.

The material properties corresponding to commonly used offshore steel (API-2H, 355D, etc.) are entered in ANSYS software.  Table 3 lists the details of material specifications for steel 355D.

Much of the information for offshore wind energy development is referenced to a proceeding paper written by Chiang et al. [1], titled “The Potential of Wave and Offshore Wind Energy in Around the Coastline of Malaysia that Face the South China Sea,” presented at the International Symposium on Renewable Energy held in Kuala Lumpur, Malaysia. In this project, the region of Sabah, Sarawak, and East Coast of Peninsular Malaysia were identified as potential sites for offshore wind platforms with the water depth up to 50 m. The annual wind speeds averaged at about 1-2 knots, reaching a maximum of 5 knots during monsoon periods.

The surface model contains the shown dimensions and will be imported into ANSYS AQWA Workbench later. However, surface model does not have mass information which ANSYS can automatically generate. The same model is recreated as a solid model and shelled to 10 mm. The resulting solid model is a real-world representation of the structure and is created so that mass and moment properties can be extracted from SolidWorks.

Autogenerated mass information based on dimension and material specification is as follows:

 Mass properties of structure solid model (part configuration: default).
 Output coordinate system: —default—.
 Density = 7850.00 kilograms per cubic meter.
 Mass = 10444.91 kilograms.
 Volume = 1.33 cubic meters.
 Surface area = 266.49 m^2^.
 Center of mass: (meters):
  (1) X=4.10,Y=2.25,Z=3.33.
 Principal axes of inertia and principal moments of inertia: (kilograms *∗* square meters).
 Taken at the center of mass:
  (2) Ix=0.88,0.48,0.00,Px=189209.00,Iy=−0.48,0.88,0.00,Py=200253.92,Iz=0.00,0.00,1.00,Pz=226763.10.
 Moments of inertia: (kilograms *∗* square meters).
 Taken at the center of mass and aligned with the output coordinate system:
  (3) Lxx=191764.93,Lxy=4658.03,Lxz=0.00,Lyx=4658.03,Lyy=197697.99,Lyz=0.00,Lzx=0.00,Lzy=0.00,Lzz=226763.10.
 Moments of inertia: (kilograms *∗* square meters).
 Taken at the output coordinate system:
  (4) Lxx=360278.82,Lxy=100960.08,Lxz=142481.33,Lyx=100960.08,Lyy=488874.48,Lyz=78181.54,Lzx=142481.33,Lzy=78181.54,Lzz=455110.32.

**Figure 8:**
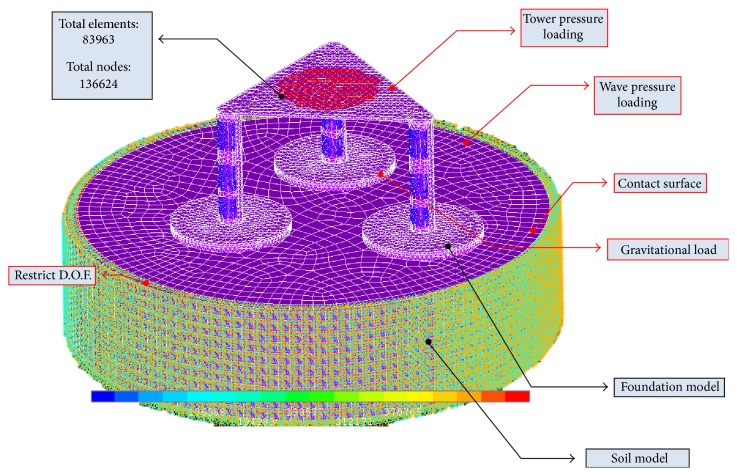
Summary of ANSYS model.

The density will be used in material specifications inside ANSYS Classic. The Center of Mass and Moment of Inertia is also useful as an input parameter in the Hydrodynamic Diffraction analysis for a more realistic approximation of the problem.  Figure 8 shows the summary of ANSYS model.

For the wave pressure loading, results from ANSYS Hydrodynamic Diffraction, which is a contour plot of the maximum pressure experienced, is used by approximating it inside the model with the highest pressure on the top, medium pressure in between, and low pressure on the bottom of the model. It is assumed that this maximum pressure will come from the same direction at once.

As for the tower loading, calculation for the pressure is as follows: NREL 5 MW turbine total mass including tower, rotor, and nacelle (approx.): 700,000 kg. By assuming the tower diameter to be approximately 5 m at the base, the pressure exerted on platform is 546548 Pa. The effect of wind loading is assumed to be insignificant and not accounted for in the model.

## 4. Results and Discussion

**Figure 9:**
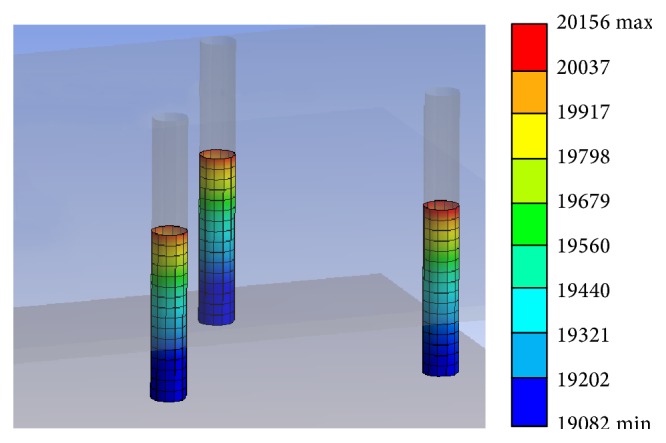
Pressure force due to wave loading.

Figure 9 shows the pressure force due to wave loading based on ANSYS AQWA Hydrodynamic Diffraction result. The result shown in Figure 7 indicates a wave frequency of 0.072 Hz with amplitude of 1 m and the pressure contour is shown in units of Pascal. As expected, the highest forces are found near the top of the structure where waves have the strongest impact. Lowering down the structure, most of the force is contributed by hydrostatic forces that increase the deeper we go. The caissons were not modeled as they are assumed to be close to the bottom of the sea and do not account for much wave/current loading.

**Figure 10:**
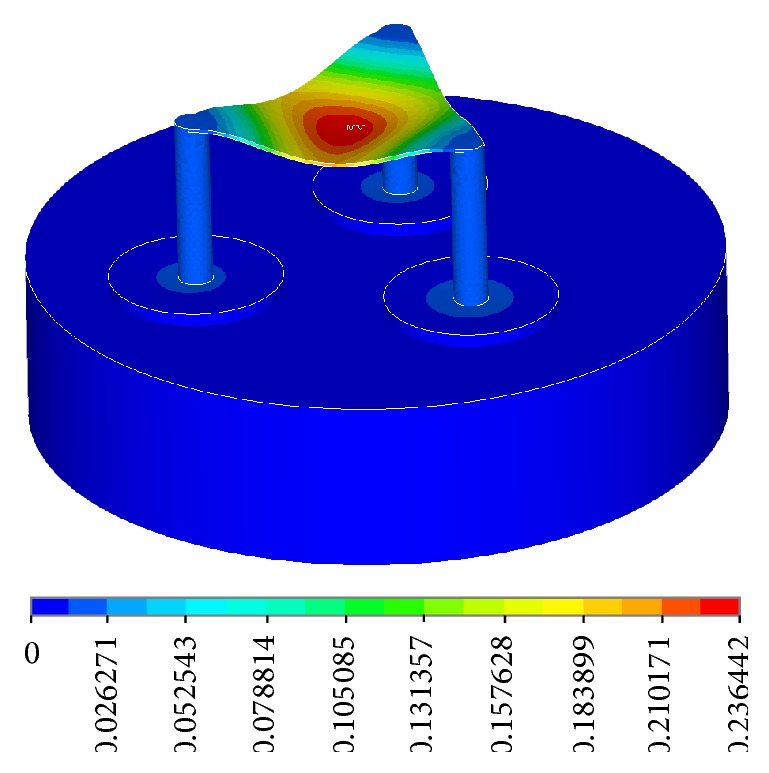
Displacement Vector Sum (Nodal Solution).

The pressure reading is given as an averaged solution from wave forces coming from all directions. Hydrostatic pressure is automatically incorporated into the solution, leading to a small difference between the pressure at the top (mainly contributed by wave forces) and the pressure at the bottom (mainly contributed by hydrostatic pressure). The total displacement vector plot indicates that the area of highest displacement comes at the center with the maximum displacement being 0.236 m, or about 25 cm (Figure 10). This is an acceptably small value given the fact that the structure is located out at sea with large wave forces acting on it. The structure shows little movement of the caisson, making it a stable structure for use. The maximum displacement is about 0.8 cm occurring near the base on the structure.

**Figure 11:**
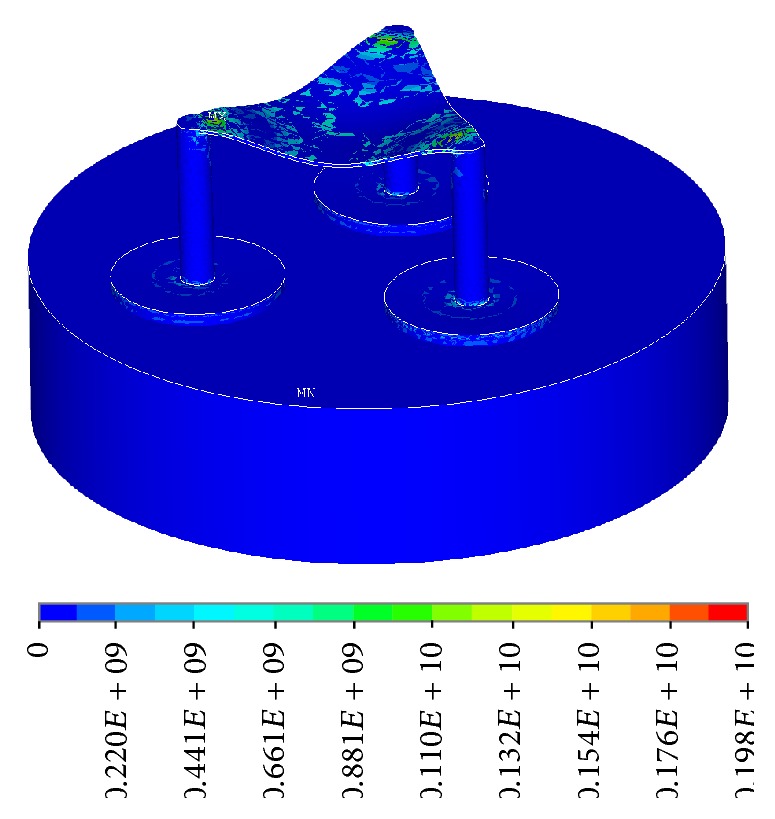
Stress intensity (Nodal Solution).

**Figure 12:**
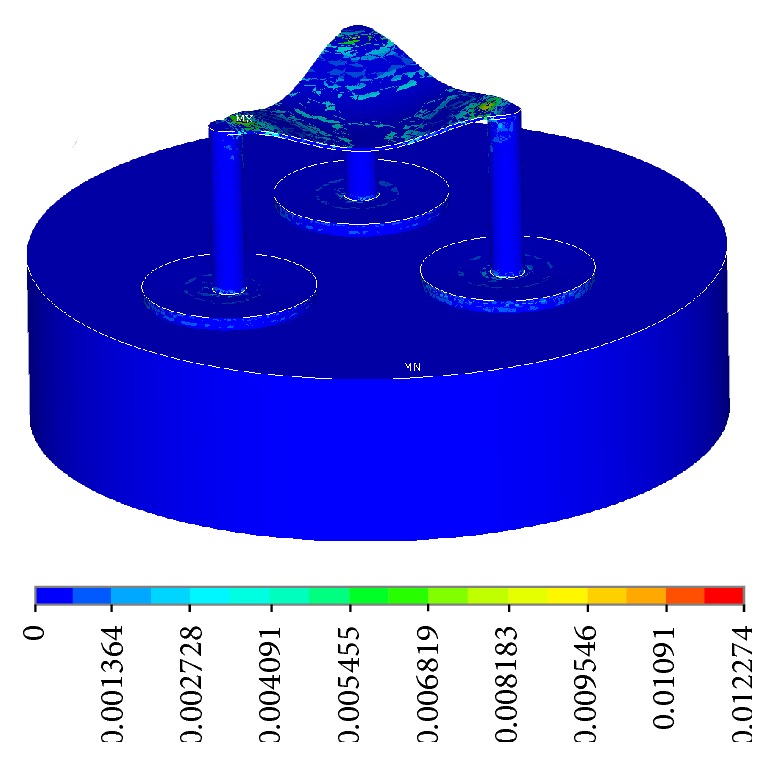
Strain intensity (Nodal Solution).

The stress intensity plots show the maximum stresses occurring at 0.198*e*10 Pascal, which is only within 25% of the tensile strength of the material at 80*e*9 Pascal, with good room for a large factor of safety (Figure 11). The plot did not show a smooth curve due to the use of coarse elements. As the elements get finer, the transitions between forces will be smoother. The strain intensity plot is similar to the stress intensity, showing a maximum strain of 0.122 (Figure 12). The following are frame-by-frame animations of the first three lowest modal shapes successfully extracted by ANSYS. Structure displacement is exaggerated to aid visualization.

**Figure 13:**
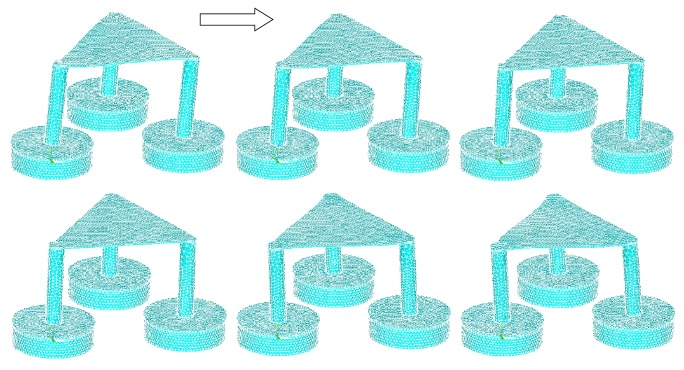
First modal shape.

**Figure 14:**
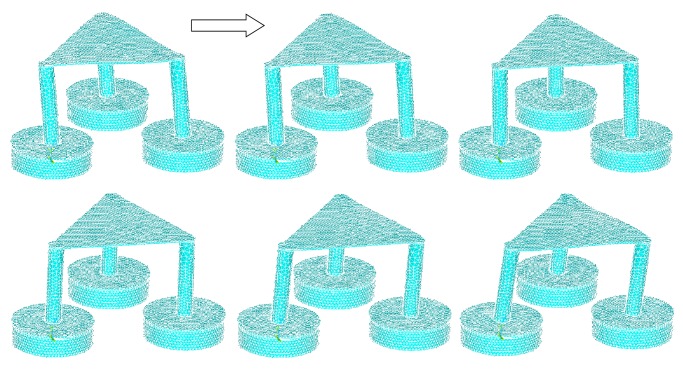
Second modal shape.

**Figure 15:**
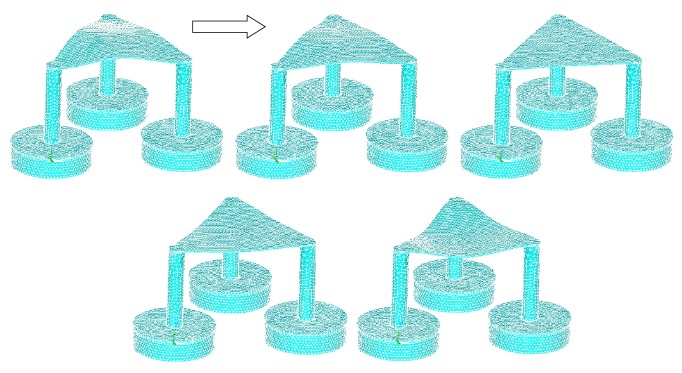
Third modal shape.

Figures 13, 14, and 15 show three generations of mode shapes were developed in order to represent the model. The following are frame-by-frame animations of the first three lowest modal shapes successfully extracted by ANSYS. Structure displacement is exaggerated to aid visualization.  Table 4 lists the modal frequency. The first mode shape occurs at 4.6 Hz, the second at 4.7 Hz, and the third at 7.5 Hz.

This information will be useful in analyzing the structure's compliance with the dynamic conditions faced at the final installation site. The first two modal shapes are important for comparing against wave induced resonance while the third is important for resonance due to turbine and rotor/blades.

The structure is modeled using a simple set of primitives. This is necessary in order to reduce the complexity of the structure and thus lower the number of elements and nodes needed to for developing the model. Based on the results, the maximum displacement occurs as expected in the middle of the structure where the wind turbine was placed on. The maximum value of displacement is 20 cm. Given the size of the structure, this is an insignificant deflection and is expected given the structure deck is simply a thick metal plate. The structure can be further reinforced with supporting structures such as welding a T-beam onto it to further strengthen the middle section should it be desirable. The *y*-component displacement is included to show the effect of wave loading on the structure. On the total displacement structure, due to the high (relatively) displacement of the middle section of the structure caused by the massive weight of the turbine and tower, the displacement by wave is nearly invisible. However, the *y*-component displacement clearly shows that there is indeed a measurable deflection of the structure as a result of the wave forces encountered.

The modal analysis results show that most of the resonant mode shapes are in the region of 3 Hz and above. This is favorable because most literature recommendations on offshore wind turbine support structure design dictate that the first resonant mode shape must be above a minimum of 0.1–0.5 Hz, depending on the actual characteristics of the turbine and sea wave conditions. Most of the resonant frequencies encountered lie below the 1 Hz limit.

## 5. Conclusion

The use of three-dimensional finite element simulation using ANSYS in designing of offshore wind power platform could participate efficiently at the design stage. The design of platform showed the mode shape generation and complied with the dynamic conditions faced at the final installation site. The result analysis determined resonance which could significantly reduce the lifespan of the structure. In the ocean where there are a lot of dynamic interactions between wind, wave, and structure, it is important that the structure's resonant frequencies lie sufficiently away from the mean resonant frequencies.

## Figures and Tables

**Table 1:** Comparison of common foundations (Source: [9]).

Type	Environmental impact	Technical benefits	Limitations
Monopile	Installation consists of hammering into seabed that can cause disturbance to local marine life.	Simple and proven design.	(i) Limited to water depths of about 10 meters. Deeper waters limit technical feasibility of manufacturing large diameter piles. (ii) Requires scour protection for very shallow waters. (iii) Unsuitable for locations with large boulders.

Suction caisson	Very little impact.	Can be used in areas of high geological soft mud.	Use depends on site conditions. Boulders on seabed limit use.

Gravity-type	Significant disturbance of seabed.	Simple and proven design.	Requires a hard seabed as well as seabed preparation.

Tripod	No significant impact.	(i) Simple and proven design. (ii) Minimal site preparation.	No major limitations besides being limited to water depths of about 30 m.

Compliant tower	No significant impact.	Suitable for deep waters; compliance reduces need for stiff structure.	Requires very accurate positioning of tendons on seabed.

Jacket	Impact on seabed during installation of jacket legs.	Suitable for deeper transitional waters.	Limited to water depths from 20 to 50 m.

**Table 2:** Gross properties chosen for the NREL 5 MW Baseline wind turbine [10].

Rating	5 MW
Rotor orientation and configuration	Upwind, 3 blades
Control	Variable speed, collective pitch
Drivetrain	High speed, multiple-stage gearbox
Rotor and hub diameter	126 m, 3 m
Hub height	90 m
Cut-in, rated, and cut-out wind speed	3 m/s, 11.4 m/s, and 25 m/s
Cut-in, rated rotor speed	6.9 rpm, 12.1 rpm
Rated tip speed	80 m/s
Overhang, shaft tilt, and precone	5 m, 5°, 2.5°
Rotor mass	110,000 kg
Nacelle mass	240,000 kg
Tower mass	347,460 kg
Coordinate location of overall CM	(−0.2 m, 0.0 m, and 64.0 m)

**Table 3:** Material specifications for Steel 355D.

Property	Value	Units
Elastic modulus	210*e* + 009	N/m^2^
Poisson's ratio	0.3	
Shear modulus	80.9*e* + 009	N/m^2^
Density	7850	Kg/m^3^
Thermal conductivity	0.2256	W/m·K
Specific heat	1386	J/kg·K
Tensile strength	80*e* + 009	N/m^2^

**Table 4:** The modal frequency.

Set	Frequency	Load step	Substep
1	4.6837	1	1
2	4.7102	1	2
3	7.5579	1	3
